# Comparison of Three Species of Rhubarb in Inhibiting Vascular Endothelial Injury via Regulation of PI3K/AKT/NF-*κ*B Signaling Pathway

**DOI:** 10.1155/2022/8979329

**Published:** 2022-03-28

**Authors:** Xin Li, Songli Huang, Bingyu Zhuo, Jingyan Hu, Yue Shi, Jiangyi Zhao, Jincheng Tang, Xiuhua Hu, Shengli Wei

**Affiliations:** ^1^School of Chinese Materia Medica, Beijing University of Chinese Medicine, Beijing 102488, China; ^2^School of Life Sciences, Beijing University of Chinese Medicine, Beijing 102488, China; ^3^Engineering Research Center of Good Agricultural Practice for Chinese Crude Drugs, Ministry of Education, Beijing 100102, China

## Abstract

**Background/Aim:**

Rhubarb, a traditional Chinese medicine derived from three species, is commonly used in the prescriptions for promoting blood circulation and removing blood stasis based on its traditional effects of removing blood stasis and dredging the meridians. It has been reported that rhubarb can protect blood vessels by reducing inflammation and inhibiting vascular endothelial injury (VEI), but the effective components and mechanism of rhubarb inhibiting VEI are still unclear. This study aimed to compare the differences in chemical compositions of the three species of rhubarb and their inhibitory effect on VEI, so as to explain the material basis and select the dominant species to inhibit VEI, and to elucidate the mechanism of rhubarb's inhibitory effect on VEI.

**Methods:**

Plant metabolomics was used to compare the chemical components of three species of rhubarb. The efficacy of three species of rhubarb in inhibiting VEI was compared through cell experiments *in vitro*. At the same time, combined with network pharmacology and molecular docking, the effective components and pathways of rhubarb involved in inhibiting VEI were screened. The mechanism of rhubarb inhibiting VEI was verified by molecular biology.

**Results:**

There were significant differences in the distribution of chemical components among the three species of rhubarb. We identified 36 different chemical components in the positive ion mode and 38 different chemical components in the negative ion mode. Subsequently, the results showed significant differences in inhibiting VEI among the three species of rhubarb based on the contents of inflammatory factors (such as IL-1*β*, IL-6, and TNF-*α*), ROS, and NO and confirmed that *R. tanguticum* had the best inhibitory effect on VEI in the light of the comprehensive efficacy, compared with *R. palmatum* and *R. officinale*. Three species of rhubarb alleviated the inflammatory response in LPS-induced EA.hy926 cells by reducing the contents of inflammatory cytokines IL-6, IL-1*β*, and TNF-*α* and decreasing expressions of PI3K, AKT, NF-*κ*B p65, and STAT3 protein in the PI3K/AKT/NF-*κ*B pathway and the inhibition of proteins phosphorylation. In addition, three species of rhubarb could lessen the contents of ROS and NO in EA.hy926 cells induced by LPS. All results indicated that the process of inflammation-induced cellular oxidative stress, which resulted in VEI, was obviously improved by three species of rhubarb.

**Conclusion:**

*R. tanguticum* was more effective among three species of rhubarb, and it had been proved that gallic acid, gallic-acid-*O*-galloyl-glucoside, procyanidin B-2,3,3′-di-*O*-gallatein, and other potential components could reduce the contents of inflammatory factors (such as IL-1*β*, IL-6, and TNF-*α*), ROS, and NO by inhibiting the PI3K/AKT/NF-*κ*B signaling pathway and protected the vascular endothelium and the blood vessels by improving the inflammation and oxidative stress reaction.

## 1. Introduction

It is a common phenomenon that a kind of traditional Chinese medicine (TCM) is derived from multiple species, such as *Coptis* [1] and licorice [2]. Meanwhile, for the most of TCM with multiple species, the differences in the types, contents, and efficacy of active ingredients among different species are still unclear, and there is no scientific explanation as to whether it is reasonable to use multiple species as one TCM. Rhubarb, derived from the roots and rhizomes of *Rheum palmatum* L. (*R. palmatum*), *Rheum tanguticum* Maxim. ex Balf.(*R. tanguticum*), and *Rheum officinale* Baill.(*R. officinale*) [3], was first recorded in “Shennong's Classic of Materia Medica” as “mainly used for lowering blood stasis and breaking up accumulation of obstruction in blood.” Nowadays, it has the effect of removing blood stasis and dredging the meridians [4], which is widely used in the field of emergency medicine and has high medicinal value [5]. Currently, the comparison of the three species of rhubarb mainly focused on properties [6], microscopy [7], chemical characteristics [8], etc., while comparative studies on pharmacological activities of three species of rhubarb were rarely reported. In addition, the indicators in the comparative study of multiple species of rhubarb were relatively single, and the methods were uneven [6, 9], and it was difficult to select the species with advantages in a certain efficacy. More importantly, the current problem with TCM research was that the studies of efficacy and chemical compositions were mostly independent of each other [10–12]. Ingredients are the basis of medicinal effects, and the correlation study between the two at the same time is more conducive to the discovery of effective ingredients and medicinal effects [13]. Therefore, it is urgent to compare the active components of different species of rhubarb and to correlate the components and functions, so as to accurately locate the functional components in different species of rhubarb.

Vascular endothelial injury (VEI) is not only an important cause of sepsis [14] and cardiovascular disease [15], but also an important inducement of blood stasis syndrome [16]. Besides, VEI would inevitably lead to the imbalance of hemagglutination-fibrinolysis system, resulting in bleeding or thrombosis. Hypoxia and inflammation are important inducements of VEI [17]. It has been reported that rhubarb had the functions of anti-inflammatory, inhibiting platelet agglutination and improving microcirculation [7], which were also the main treatment methods of relieving VEI [18, 19]. Simultaneously, VEI was an important pathological basis for inducing blood stasis syndrome [16], and rhubarb could be used to treat blood stasis syndrome [4]. Hence, whether rhubarb with the effect of “removing blood stasis and dredging the meridians” can take effect on VEI and its mechanism remains to be further studied. In this paper, based on the methods of plant metabolomics, network pharmacology, molecular docking, and cell experiment *in vitro*, the biological effect and material basis of three species of rhubarb on VEI were systematically investigated, which also confirmed the intrinsic ways and dominant species of rhubarb inhibiting VEI.

## 2. Materials and Methods

### 2.1. Materials and Reagents

LC-MS-grade acetonitrile were purchased from Fisher Scientific (Pittsburgh, PA, USA). Purified water from a Milli-Q purification system (Millipore, Bedford, MA, USA) was used throughout the experiments. All other chemicals were of reagent grade.

The rhubarb used in the experiment was collected from the rhubarb experiment base in Tuanjie Village (Axi Town, Zoige County, Aba Prefecture, Sichuan Province, China). The samples were identified by Professor Wei Shengli of Beijing University of Traditional Chinese Medicine. The numbers a1-a8 were *R. palmatum*, the numbers t1-t12 were *R. tanguticum*, and the numbers o1-o9 were *R. officinale*. The roots of rhubarb with a diameter of more than 1 cm were cross-cut into 1-cm-thick sections. Dried raw material was crushed and sifted by 65 mesh sieve. The voucher specimens were deposited in the Resource Laboratory of Chinese Medicine, Beijing University of Chinese Medicine.

### 2.2. Sample Preparation

A precise 1.00 g rhubarb powder was put into an Erlenmeyer flask with 25 mL methanol and then weighed them (*w*_1_). It took 30 minutes to extract the active ingredients by ultrasonic wave with 40 kHz. After ultrasonic extraction, weigh again (*w*_2_) when the alcohol extract was cold. If *w*_1_ was greater than *w*_2_, add methanol to the alcohol extract to make it equal. Then the alcohol extract was filtered through a microporous membrane with a diameter of 0.22 microns. According to the previous cell viability experiment, the IC_25_ of *R. officinale* was selected as the medium dose group of three species of rhubarb to inhibit VEI, and the 1/2IC_25_ and 2IC_25_ were administered as the low- and high-dose groups, respectively. Hence, the extract was dissolved in PBS at various concentrations (132.8, 265.5, and 531 *μ*g/mL) for cell experiments.

### 2.3. Analysis of Chemical Constituents of Three Species of Rhubarb

#### 2.3.1. Instrumentation

Dionex UltiMate 3000 high-pressure liquid chromatography (Thermo Fisher Scientific, USA) was used for component separation. An ACQUITY BEH C_18_ column (2.1 × 50 mm, 1.7 *μ*m, Waters, Milford, MA, USA) was used with 30°C. The mobile phase consisted of acetonitrile with 0.1% formic acid (A) and acetonitrile (B) at a flow rate of 0.21 mL/min. The gradient elution procedure was as follows: 0-0.5min, 7%-10% B; 0.5-5.5min, 10%-15% B; 5.5-6.5min, 15%-15.1% B; 6.5-7.5min, 15.1%-15.2% B; 7.5-9.5min, 15.2%-15.3% B; 9.5-10min, 15.3%-15.5% B; 10-13min, 15.5%-17% B; 13-14min, 17% B; 14-15min, 17%-20% B; 15-17min, 20%-25% B; 17-20min, 25%-28% B; 20-25min, 28%-35% B; 25-26min, 35%-41% B; 26-27.5min, 41%-90% B; 27.5-29.5min, 90%-7% B; and 29.5-30min, 7% B. The injection volume was 0.4 *μ*L. The detection wavelength was 230 nm.

The LC system was coupled to a Q-Exactive Orbitrap High-Resolution Mass Spectrometer (Thermo Fisher Scientific, USA). The mass spectrometer was operated both in positive and negative ion mode with HESI source. The parameters were as follows: N_2_; 3.8 KV (positive ion mode) and 3 KV (negative ion mode); ion source temperature 350°C; capillary voltage, 320°C; sheath gas (nitrogen) flow, 40arb; aux gas flow, 10arb; and full-scan spectra mass range, 100~1500 Da.

#### 2.3.2. Data Processing and Multivariate Analysis

The raw mass data were analyzed with the Statistical Iterative Exploratory Visualization Environment (SIEVE 2.2) for peak extraction, alignment, and visualization. The data-filtered conditions were as follows: retention time was 0~30 min, the specific mass to charge ratio *(m/z*) was 100~1500, frame time width was 2.50, and *m/z* width was 10 ppm. MetaboAnalyst 4.0 (https://www.metaboanalyst.ca/) was used to standardize data. Multivariate analysis was realized by introducing the resultant data to Simca14.0, and principle component analysis (PCA) and orthogonal projection to latent structures-discriminant analysis (OPLS-DA) were used to find the different components among the rhubarb samples with three species. The conditions for becoming a difference component were VIP >1 in OPLS-DA analysis and *P* < 0.05 in one-way analysis of variance, which represented a significant difference in statistics. Then infer the structural formula information of the different components through the HMDB (http://www.hmdb.ca/) and METLIN (http://metlin.Scripps.edu/) databases.

### 2.4. Inhibitory Effects of Three Species of Rhubarb on VEI

#### 2.4.1. Cell Culture

EA.hy926 cells were purchased from the National Biomedical Experimental Cell Resource Bank (Beijing, China). EA.hy926 cells were cultured in DMEM medium containing 10% fetal bovine serum and 1% penicillin/streptomycin at 37°C, 5% CO_2_. The experiment was carried out with cells on logarithmic growth phase.

#### 2.4.2. Cell Viability Assay

EA.hy926 cells were, respectively, treated with lipopolysaccharides (LPS, #L2880, Sigma, USA) at various concentrations (0.001-1000 *μ*g/mL) for 24 h or the extracts from three species of rhubarb (132.8, 265.5, 531 *μ*g/mL) for 48 h in 96-well plates (4.5 × 10^3^ cells). After the medium was removed, CCK8 (#MA0218, Meilunbio, China) was added and incubated for another 1 h and then determined the absorbance at 450 nm.

#### 2.4.3. Enzyme-Linked Immunosorbent Assay (ELISA)

EA.hy926 cells were treated with LPS (1 *μ*g/mL) for 24 h before the addition of extracts from three species of rhubarb (132.8, 265.5, 531 *μ*g/mL) for 48 h in 24-well plates. The supernatant of the medium was collected and centrifugated. The contents of TNF-*α* (#KE00154), IL-6 (#KE00139), and IL-1*β* (#KE00021) in the supernatant were determined according to the procedure of the kit (Proteintech, USA).

#### 2.4.4. Detection of NO Content

EA.hy926 cells were treated with LPS (1 *μ*g/mL) for 24 h before the addition of extracts from three species of rhubarb (132.8, 265.5, 531 *μ*g/mL) for 48 h in 24-well plates. The supernatant of the medium was collected and centrifugated. The contents of NO in the supernatant were determined according to the manufacturer's instructions (Nanjing, China).

#### 2.4.5. Detection of ROS

2′,7′-Dichlorodihydrofluorescein diacetate (DCFH-DA, #HY-D0940, MCE, USA) was used to detect the contents of ROS in cells. Extracts from three species of rhubarb (132.8, 265.5, 531 *μ*g/mL) were added in EA.hy926 cells treated by LPS (1 *μ*g/mL). After 48-h incubation, the cells were treated with 10 *μ*M DCFH-DA and 8 *μ*M Hoechst 33342 (#C0030-10, Solarbio, China) for 30 min at 37°C, then washed the wells with PBS three times, and investigated the ROS by confocal microscopy (Olympus, Japan).

### 2.5. Screening Effective Components of Three Species of Rhubarb Inhibiting VEI by Component-Pharmacodynamic Correlation Analysis

The combined efficacy values (I) of TNF-*α*, IL-6, IL-1*β*, NO, and ROS were calculated by formulas (1) and (2). The gray correlation was applied to analyze the relative correlation between the different chemical components of three species of rhubarb and the comprehensive efficacy value. The components with correlation degree greater than 0.7 were selected as the potential effective components of three species of rhubarb in inhibiting VEI.

(1) $$\begin{array}{c} \text{The combined efficacy values }\left(I\right)=A*D^{\prime }, \end{array}$$

where *A* is the content of index (TNF-*α*, IL-6, IL-1*β*, NO, and ROS) and *D*′ is the weight of index (TNF-*α*, IL-6, IL-1*β*, NO, and ROS)

(2) $$\begin{array}{c} D^{\prime }=\frac{D_{i}}{\sum_{1}^{5}D_{i}}, \end{array}$$

where *Di* is the sum of the measured values of an index in all treatment groups and *i* = 1, 2, 3 ⋯ ⋯, 5.

### 2.6. Network Pharmacology and Molecular Docking

What is the mechanism of rhubarb inhibiting VEI? Network pharmacology combined with molecular docking were used to predict the mechanism. We calculated the target 1 of three species of rhubarb through SuperPred and SwissTargetPrediction database (C-T network) and calculated the target 2 of “vascular inflammation” through GeneCard, OMIM, and DisGeNET database (T-D network). The common targets of target 1 and target 2 were the potential targets of rhubarb in inhibiting VEI. DAVID database was used to analyze the enrichment of GO and KEGG pathways, and then the “components-targets-pathways” network of rhubarb inhibiting VEI was constructed. STRING database was used to construct the protein-protein interaction (PPI) network, and the top 20 targets with the highest connectivity were screened for molecular docking with the active components using Discovery Studio 2019.

### 2.7. Western Blotting

Extracts from three species of rhubarb (132.8, 265.5, 531 *μ*g/mL) were added in EA.hy926 cells treated by LPS (1 *μ*g/mL). After 48-h incubation, the cells were lysed in RIPA buffer containing the mixture of protease inhibitor and PMSF(#C1055, Beijing, China), and homogenates were centrifuged at 12000 rpm for 5 min at 4°C, and then the supernatant was harvested. According to the protocol, the protein denaturation was carried out after the protein concentration was determined. Equal amounts of protein were subjected to 8% SDS-PAGE and transferred to PVDF membrane. The membrane was incubated with anti-p65, anti-AKT, and anti-STAT3 (1:1500, Proteintech, USA); anti-p-p65 and anti-p-PI3K(1:1000, CST, USA); anti-PI3K, anti-eNOS, and anti-iNOS (1:1000, Proteintech, USA); anti-p-AKT(1:2000, Proteintech, USA); and anti-p-STAT3 (1:2000, Abcam, USA), at 4°C overnight, and then the membrane was incubated with secondary antibody and finally added chemiluminescence solution for development.

### 2.8. Immunofluorescence

The nuclear translocation of p65 and STAT3 was observed by immunofluorescence technique. Extracts from three species of rhubarb (132.8, 265.5, 531 *μ*g/mL) were added in EA.hy926 cells treated by LPS (1 *μ*g/mL). After 48-h incubation, 1 mL paraformaldehyde (4%) was added and fixed at room temperature for 30 min. And then 1 mL Triton X-100 solution (0.1%) was added and permeated for 20 min. After the Triton X-100 solution was discarded, 1 mL BSA blocked for 1 h. Then, they were washed with PBS and incubated with p65 primary antibody (1:100 dilution) and STAT3 primary antibody (1:100 dilution), respectively, at 4°C overnight and, next, incubated with red fluorescent-labeled secondary antibody (1:200 dilution) at room temperature in the dark for 1 h. After maintaining DAPI (1 *μ*g/mL) for 5 min, we investigated p65 and STAT3 by confocal microscopy.

### 2.9. Statistical Analysis

All results were presented as the mean ± SD. Study data were analyzed using one-way analysis of variance (ANOVA) for significance comparison. *P* < 0.05 was considered as statistically significant.

## 3. Results

### 3.1. Analysis of Different Components in Three Species of Rhubarb

**Figure 1:**
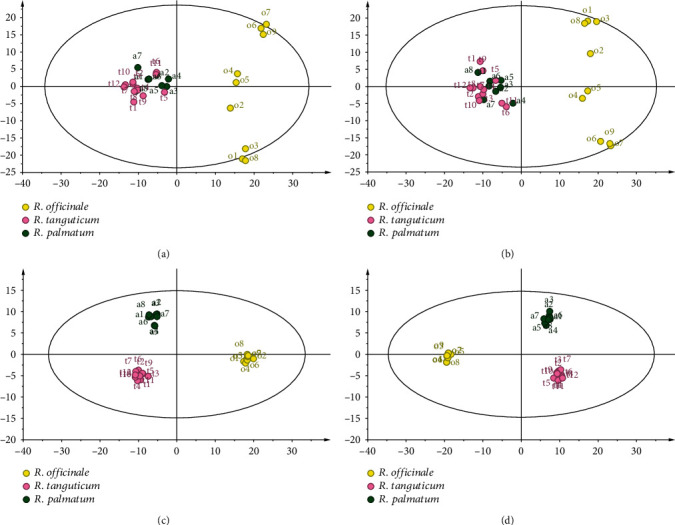
PCA and OPLS-DA diagram of three species of rhubarb: PCA in positive ion mode (a), PCA in negative ion mode (b), OPLS-DA in positive ion mode (c), and OPLS-DA in negative ion mode (d).

The model stability (*R*^2^*X*) of PCA in the positive ion mode was 0.662, and the model prediction rate (*Q*^2^) was 0.335. In negative ion mode, the model stability (*R*^2^*X*) of PCA was 0.683, and the model prediction rate (*Q*^2^) of PCA was 0.397 (Figure 1). The results of PCA showed that the three species of rhubarb could be well distinguished under positive and negative ion modes, indicating that the chemical components of the three species of rhubarb were significantly different.

OPLS-DA could be used to further search for different components on the basis of PCA analysis. In the scatter diagram of three species of rhubarb (in negative ion mode, *R*^2^*X* = 0.63, *R*^2^*Y* = 0.988, and *Q*^2^ = 0.796; in the positive ion mode, *R*^2^*X* = 0.611, *R*^2^ = 0.988, and *Q*^2^ = 0.764), it could be seen that the three species of rhubarb were obviously divided into three categories, and the discrimination of the three species of rhubarb was higher than that of PCA analysis. According to the VIP >1 of the OPLS-DA model and the Kruskal-Wallis test (*P* < 0.05), metabolites with different expression of three species of rhubarb were screened. The different chemical components were identified by METLIN, HMDB, MassBank, and other databases. A total of 36 differential chemical components were found in the positive ion mode and 38 in the negative ion mode (Tables 1 and 2).

### 3.2. Effects of LPS and Three Species of Rhubarb on the Viability of EA.hy926 Cells

**Figure 2:**
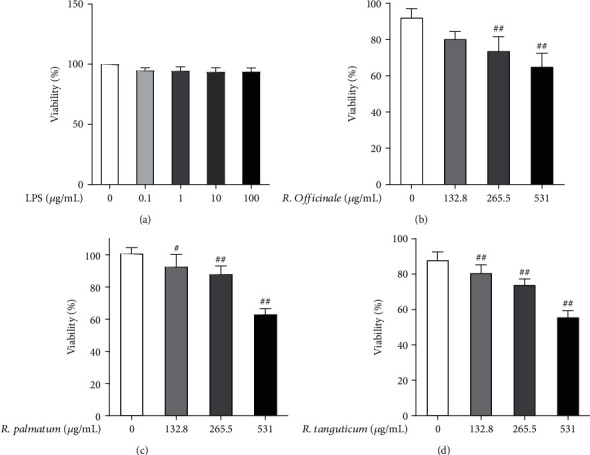
The effects of LPS and three species of rhubarb on the viability of EA.hy926 cells: LPS (a), *R. officinale* (b), *R. palmatum* (c), and *R. tanguticum* (d). Statistical significance is labeled as below: compared with the control group, #*P* < 0.05; ##*P* < 0.01.

The results of CCK8 showed that 0~100 *μ*g/mL LPS had no significant effect on the viability of EA.hy926 cells (Figure 2(a)). The doses of *R. officinale* 1/2IC_25_ (132.8 *μ*g/mL), IC_25_ (265.5 *μ*g/mL), and 2IC_25_ (531 *μ*g/mL) were used as the low-, medium-, and high-dose groups of the three species of rhubarb, respectively. After treating EA.hy926 cells for 48 h, CCK8 experimental results indicated that, the low-, medium-, and high-dose groups of *R. palmatum*, *R. tanguticum*, and *R. officinale*, all could affect the viability of EA.hy926 cells in a concentration-dependent manner (Figures 2(b)–2(d)).

### 3.3. Effects of Three Species of Rhubarb on the Inflammatory Factors of EA.hy926 Cells Induced by LPS

**Figure 3:**
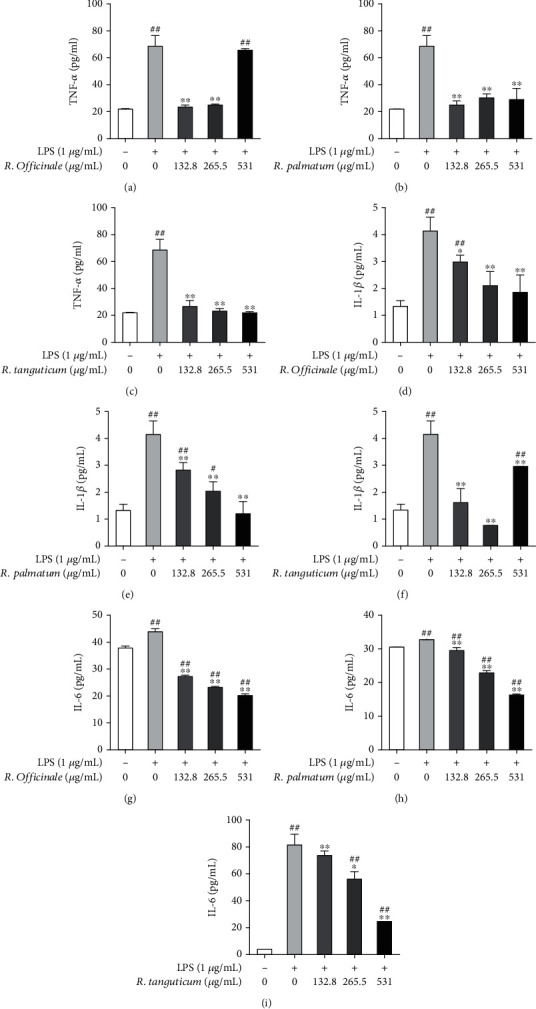
The effect of three species of rhubarb with different concentrations on the release of inflammatory factors in EA.hy926 cells induced by LPS. The content of TNF-*α* in *R. officinale* (a), *R. palmatum* (b), and *R. tanguticum* (c). The content of IL-1*β* in *R. officinale* (d), *R. palmatum* (e), and *R. tanguticum* (f). The content of IL-6 in *R. officinale* (g), *R. palmatum* (h), and *R. tanguticum* (i). Statistical significance is labeled as below: compared with the control group, #*P* < 0.05; ##*P* < 0.01. Compared with the model group, ∗*P* < 0.05; ∗∗*P* < 0.01.

When cells are stimulated by foreign substances, the immune system in the body will be activated, and the secretion of inflammatory factors, such as TNF-*α*, IL-6, and IL-1*β* will be promoted, which will directly damage endothelial cells. The results showed that the secretion of TNF-*α*, IL-1*β*, and IL-6 in the supernatant of EA.hy926 cells was significantly increased after being stimulated by 1 *μ*g/mL LPS for 24 h, and there was a significant difference between the normal control group and the model group (*P* <0.01). After treating with different concentrations (132.8 *μ*g/mL, 265.5 *μ*g/mL, and 531 *μ*g/mL) of *R. palmatum*, *R. tanguticum*, and *R. officinale*, the secretion of TNF-*α*, IL-1*β*, and IL-6 in each group had different degrees of inhibition (Figure 3). The inhibition of IL-6 secretion by three species of rhubarb showed a good dose-dependent. At the concentration of 265.5 *μ*g/mL, the three species of rhubarb had a significant inhibitory effect on the secretion of TNF-*α*, IL-1*β*, and IL-6.

### 3.4. Effects of Three Species of Rhubarb on NO of EA.hy926 Cells Induced by LPS

**Figure 4:**
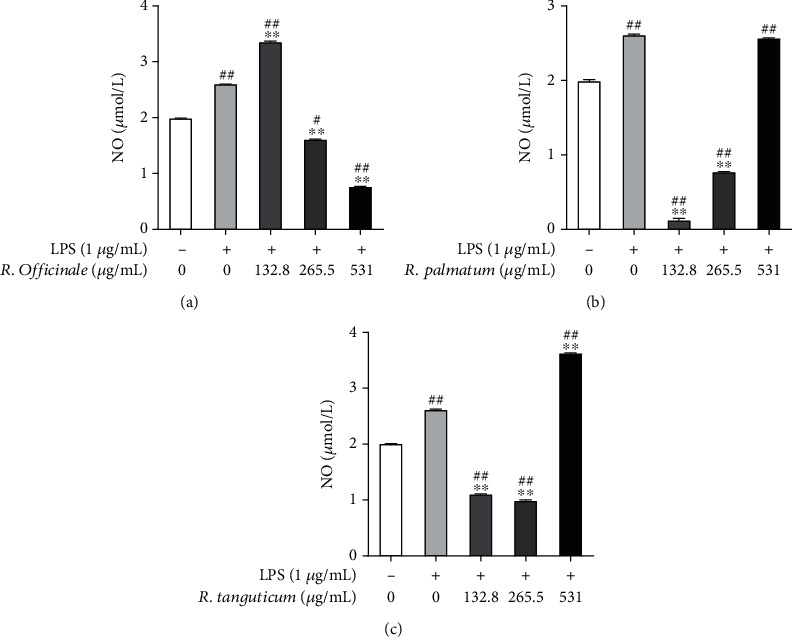
The effect of three species of rhubarb with different concentrations on the release of NO in EA.hy926 cells induced by LPS: *R. officinale* (a), *R. palmatum* (b), and *R. tanguticum* (c). Statistical significance is labeled as below: compared with the control group, #*P* < 0.05; ##*P* < 0.01. Compared with the model group, ∗*P* < 0.05; ∗∗*P* < 0.01.

The massive production of NO is one of the important signs of cellular inflammatory response. After being stimulated with 1 *μ*g/mL LPS for 24 h, the release of NO in the supernatant of EA. hy926 cells was significantly increased compared with the normal control group (*P* < 0.01). After treating with different concentrations (132.8 *μ*g/mL, 265.5 *μ*g/mL, and 531 *μ*g/mL) of three species of rhubarb, the release of NO in each group decreased to varying degrees (Figure 4). Among them, at the concentration of 265.5 *μ*g/mL, three species of rhubarb had significant inhibitory effects on the release of NO in EA.hy926 cells induced by LPS, and the *R. palmatum* group at 265.5 *μ*g/mL had the best inhibitory effect on NO release (Figure 4(b)).

### 3.5. Effects of Three Species of Rhubarb on ROS of EA.hy926 Cells Induced by LPS

**Figure 5:**
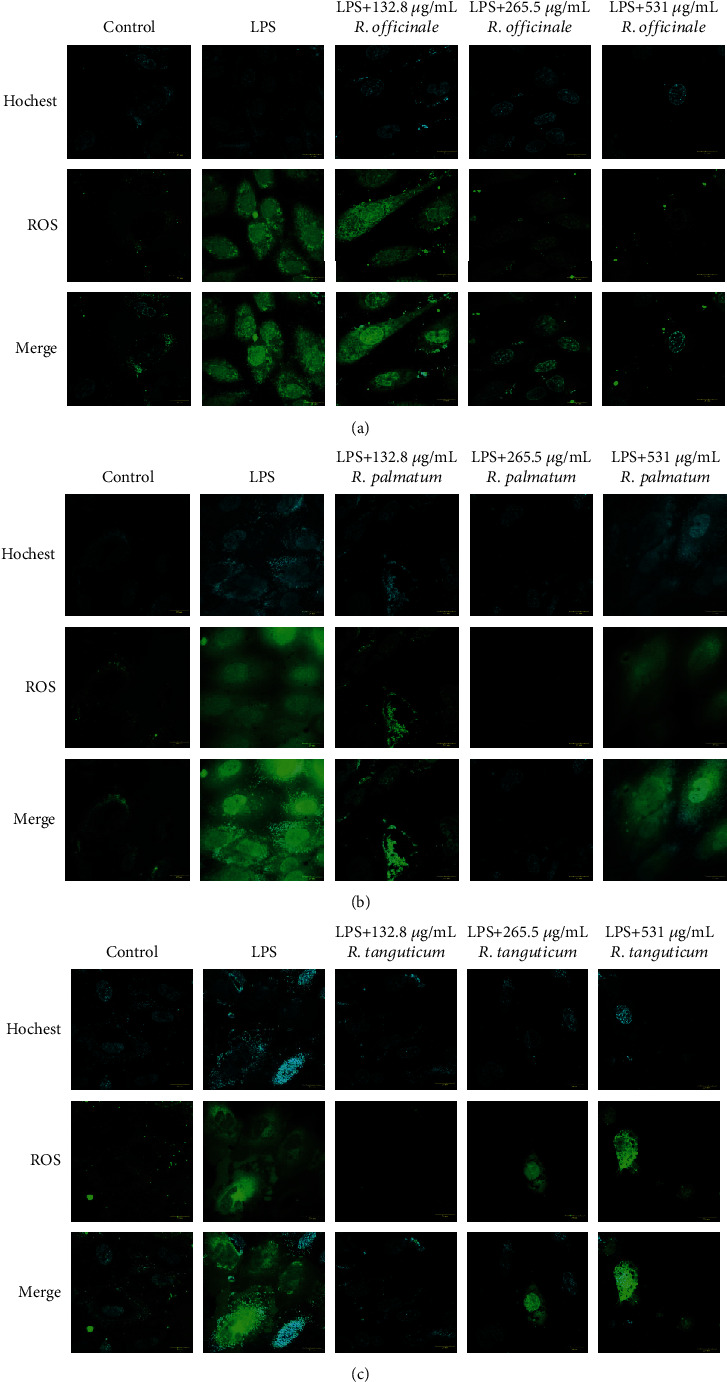
The effect of three species of rhubarb with different contents of ROS in EA.hy926 cells induced by LPS: *R. officinale* (a), *R. palmatum* (b), and *R. tanguticum* (c).

ROS is an important product of cellular oxidative stress response, which can cause irreversible damage to cell membrane. The ROS content in EA.hy926 cells was significantly increased after being stimulated with 1 *μ*g/mL LPS for 24 h. After treating with different concentrations (132.8 *μ*g/mL, 265.5 *μ*g/mL, and 531 *μ*g/mL) of three species of rhubarb, ROS content in each group showed a decreasing trend to different degrees (Figure 5). The ROS content in EA.hy926 cells induced by LPS was decreased by three species of rhubarb at 132.8 *μ*g/mL and 265.5 *μ*g/mL.

### 3.6. Screening of Potential Effective Components of Rhubarb in Inhibiting VEI

Different concentrations of three species of rhubarb (132.8 *μ*g/mL, 265.5 *μ*g/mL, and 531 *μ*g/mL) showed different inhibitory effects on NO, ROS, TNF-*α*, IL-6, and IL-1*β* in EA.hy926 cells induced by LPS. It is necessary to comprehensively consider the five indexes measured in each treatment group and finally obtain the dominant species and its appropriate dose. The comprehensive efficacy of groups with different rhubarb treatment was calculated according to formulas (1) and (2)(Table 3). The smaller the comprehensive efficacy value was, the smaller the inflammatory factors and oxidative stress response indicators were, which represented the stronger inhibitory effect of this group on VEI. The medium-dose group of *R. tanguticum* had the best effect, second was the medium-dose group of *R. palmatum*, and the effect of low-dose group of *R. officinale* was not obvious, but the inhibition of VEI in *R. officinale* groups showed a good dose-dependent effect. After grey correlation analysis was conducted between the different chemical components in three species of rhubarb and the comprehensive efficacy value, 46 potential effective components in rhubarb inhibiting VEI were screened out (Table 4). Among them, gallic acid [20] and anthocyanin [21] were consistent with the results reported in the literature.

### 3.7. Construction of Rhubarb Inhibiting VEI Network

**Figure 6:**
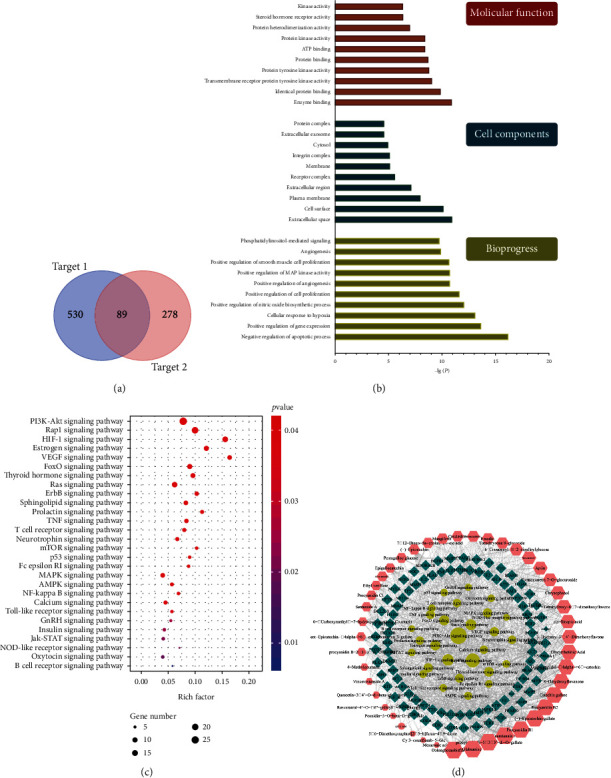
Network pharmacology analysis of rhubarb inhibiting VEI: target analysis of rhubarb inhibiting VEI (a); the enrichment analysis in biological processes, cellular components, and molecular functions of 89 identified target proteins by DAVID database (b); the enrichment analysis of KEGG by DAVID database (c); the component-target-pathway network (d).

Network pharmacology combined with molecular docking were used to predict the mechanism of 46 active components in rhubarb inhibiting VEI. The 46 potential active ingredients of rhubarb had a total of 530 ingredient targets 1 in the SuperPred and SwissTargetPrediction databases, and a total of 278 disease targets 2 in the GeneCard, OMIM, and DisGeNET gene databases have been obtained, then 530 targets 1 mapped to 278 targets 2, and finally obtained 89 potential targets for rhubarb related to VEI (Figure 6(a)). DAVID database was used to conduct enrichment analysis of GO and KEGG pathways of 89 potential targets of rhubarb in reducing VEI. According to the significance, the pathways with *P* < 0.05 were selected. GO analysis showed that the number of genes enriched in cell biological processes was relatively large, and the *P* value was low, indicating that rhubarb played a role in inhibiting VEI mainly by regulating the following biological processes (Figure 6(c)). Moreover, 309 bioprogress (e.g., negative regulation of apoptotic process, positive regulation of gene expression, cellular response to hypoxia, positive regulation of nitric oxide biological process, etc.), 40 cell components (e.g., extracellular space, cell surface, plasma membrane, etc.), and 75 molecular functions (enzyme binding, identity protein binding, transmembrane receiver protein tyrosine kinase activity, etc.) were selected. The results were shown in Figure 6(b)(sorted from small to large by *P* value, with the top 10).

KEGG analysis showed that a total of 30 pathways were enriched, including PI3K/AKT, TNF, MAPK, NF-*κ*B, and p53 signaling pathway. These classical signaling pathways played an important role in rhubarb's inhibition of VEI. According to the number of potential targets contained in the pathways, 30 pathways were sorted and visualized (Figure 6(c)). In Cytoscape 3.6.1 software, we constructed a “component-target-pathway” network diagram of rhubarb's inhibitory effect on VEI (Figure 6(d)), which could directly observe the related effects of rhubarb active components, VEI-related targets and pathways. The same active component of rhubarb could connect to different targets, and the same target could also have related effects with different active components, which once again showed that Chinese medicine was a complex system of “multicomponent, multitarget, and multipathway.” The mechanism of rhubarb inhibiting VEI was correlated with gene expression, apoptosis, protein binding, NF-*κ*B signaling pathway, and PI3K/AKT signaling pathway, which could reveal the characteristics of rhubarb inhibiting VEI from different perspectives.

**Figure 7:**
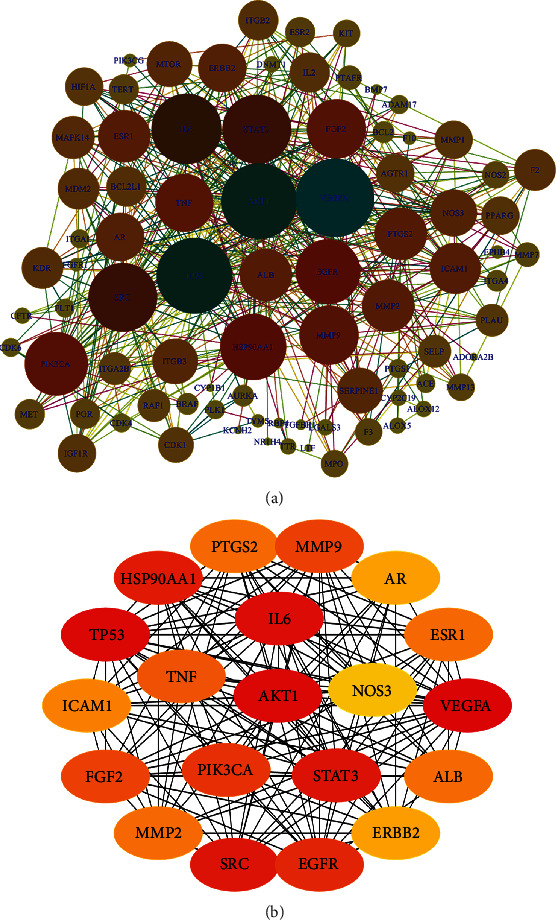
Protein-protein interactions network: protein-protein interactions between the 89 gene-coded proteins (a); protein-protein interactions between the top 20 of degrees (b).

The protein network interaction of potential targets of rhubarb inhibiting VEI was analyzed by string, and the network topology was analyzed by Cytoscape. 83 nodes and 6806 edges were obtained with an average degree of 12.4(Figure 7(a)). The top 20 proteins were screened according to the connectivity value (Figure 7(b)). Among them, VEGFA, AKT1, TP53, IL6, SRC, STAT3, and other core targets had strong interactions with other proteins in the PPI network.

Discovery Studio2019 software was used to perform molecular docking between 46 potential active components of rhubarb for inhibiting VEI and the core proteins with the top 20 connectivity values in the PPI network. The higher the molecular docking scored, the more stable the ligand and receptor bound.

**Figure 8:**
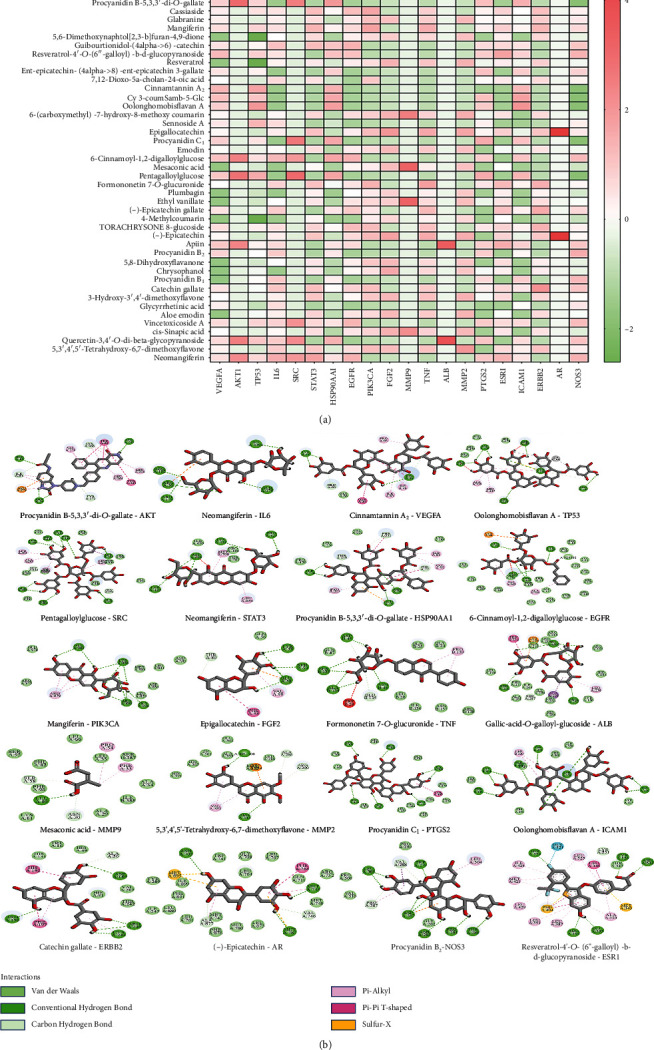
Schematic diagram of molecular docking between proteins and active components: heat map (a) and molecular docking connection diagram (b) (red represented a high relative scoring value, and green represented a low relative scoring value).

In the results of molecular docking (Figure 8(a)), the more red represents the higher the relative score, and the more green represents the lower the relative score. Each active component had a relatively high score with the target receptor protein, indicating that the active component had a good structural match with the target protein receptor. Among them (Figure 8), procyanidin B-5,3,3′-di-*O*-gallate and AKT, mangiferin and PIK3CA, neomangiferin and IL-6, formononetin 7-*O*-glucuronide and TNF, and procyanidin B_2_ and NOS_3_ score were higher(Figure 8). These components in rhubarb might act on the targets to inhibit VEI.

### 3.8. Inhibition of PI3K/AKT/NF-*κ*B Pathway and Related Protein Expression by Three Species of Rhubarb

#### 3.8.1. Inhibition of PI3K/AKT Pathway by Three Species of Rhubarb

Although we screened 46 effective components of three species of rhubarb inhibiting VEI, these were still too many. Compared with the verification of the mechanism of monomer, the extract of rhubarb was more convincing to verify the mechanism of rhubarb inhibiting VEI.

**Figure 9:**
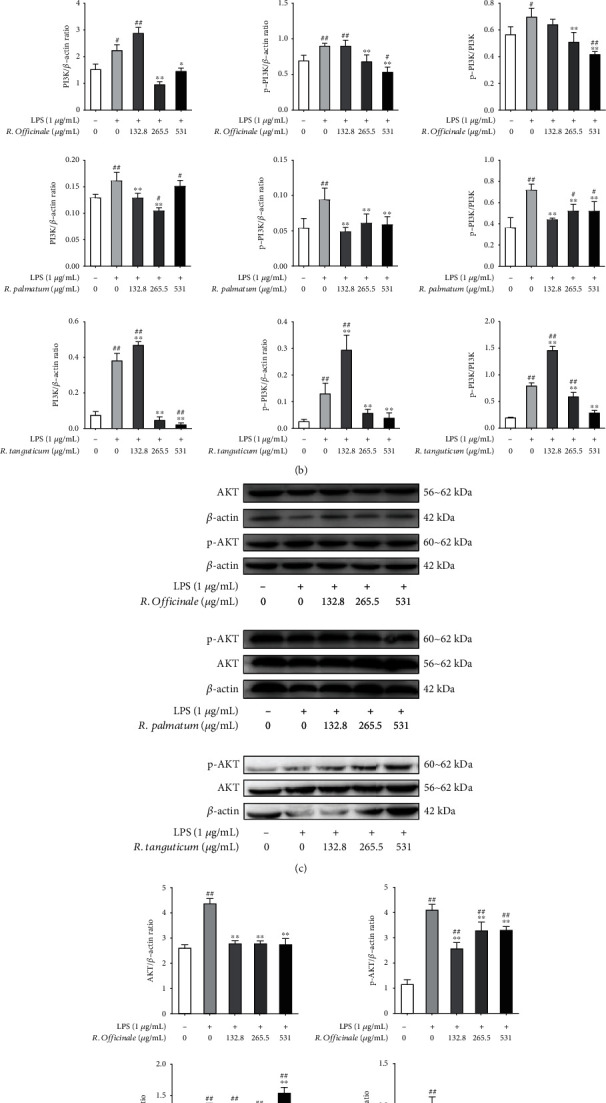
The inhibition of PI3K/AKT pathway in LPS-induced EA.hy926 cells treated with three species of rhubarb: PI3K and p-PI3K (a and b) and AKT and p-AKT (c and d).

PI3K are a group of signaling transduction enzymes, and AKT is the direct target protein of PI3K. Phosphorylation of PI3K and AKT can activate PI3K/AKT pathway, which plays an important role in the pathogenesis of inflammation, obesity, tumor, and immune diseases [22]. Moreover, PI3K/AKT pathway was also enriched by network pharmacology. In order to explore the effects of three species of rhubarb on PI3K/AKT pathway, the protein expressions of PI3K, AKT, p-PI3K, and p-AKT were detected by western blot. The results showed that LPS could increase the protein expressions of PI3K, AKT, p-PI3K, and p-AKT in EA.hy926 cells, and the protein expressions were significantly decreased (*P* < 0.05) after treated with three species of rhubarb. The difference was statistically significant (Figure 9).

#### 3.8.2. Inhibition of NF-*κ*B Pathway by Three Species of Rhubarb

**Figure 10:**
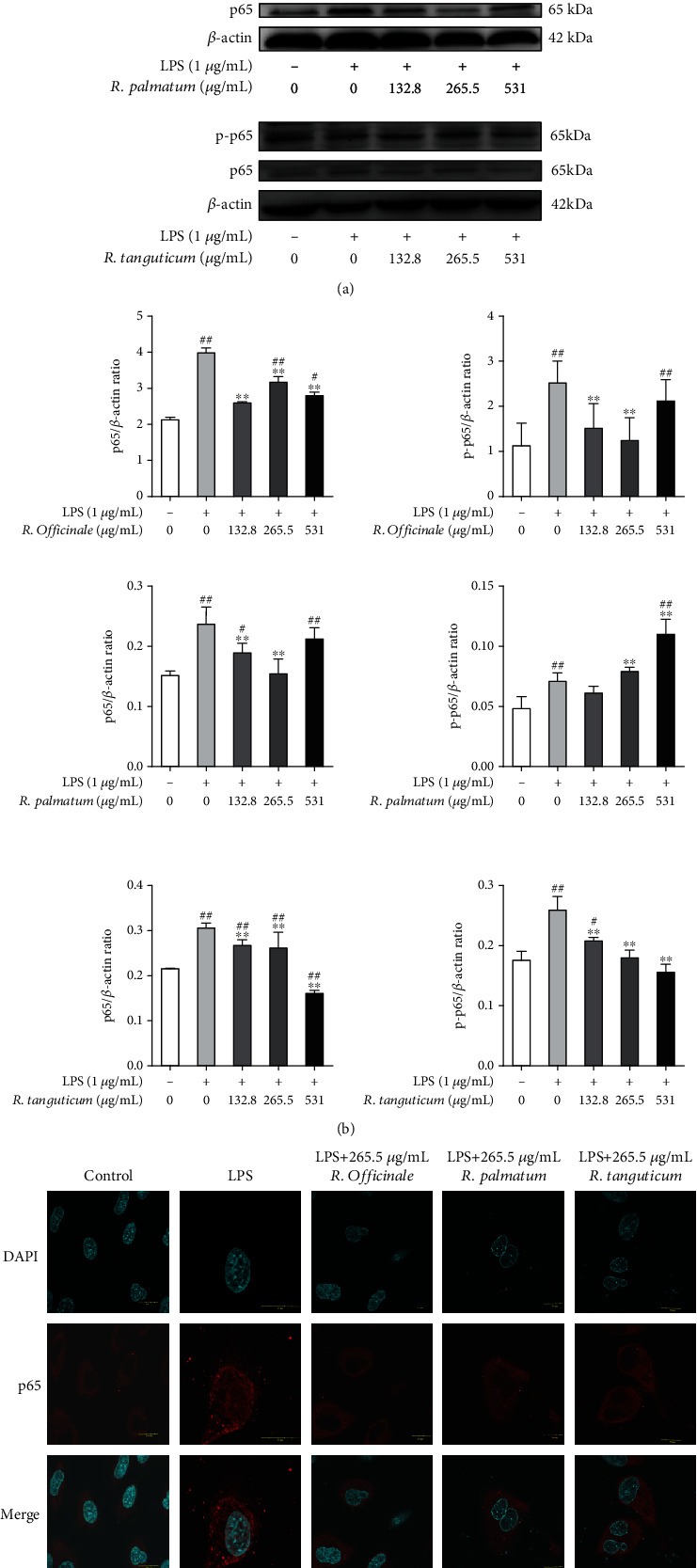
The inhibition of NF-*κ*B pathway in LPS-induced EA.hy926 cells treated with three species of rhubarb: western blot (a and b) and immunofluorescence (c).

NF-*κ*B is a transcriptional protein with multidirectional regulation, which usually enters the nucleus after phosphorylation. It can regulate the expression of a variety of inflammatory and immune genes and participate in the gene regulation of a variety of physiological and pathological processes such as inflammatory immune cell proliferation and apoptosis. It has an important impact on the pathogenesis of a series of inflammatory diseases involving cytokines and inflammatory mediators. Similarly, as a pathway predicted by network pharmacology, we used western blot to detect the protein expressions of NF-*κ*B p65 and p-p65 and used immunofluorescence assay to detect NF-*κ*B p65 phosphorylated nuclear translocation in LPS-induced EA.hy926 cells. The results of western blot showed that three species of rhubarb could significantly reduce the protein expression levels of NF-*κ*B p65 and p-p65 (*P* < 0.01)(Figure 10). Immunofluorescence assay showed that NF-*κ*B p65 was normally distributed in the cytoplasm but accumulated in the nucleus after LPS-induced phosphorylation of NF-*κ*B p65. Moderate doses of the three species of rhubarb treatments eliminated the accumulative effect of LPS-induced phosphorylation of NF-*κ*B p65 into the nucleus (Figure 10). Both results demonstrated that three species of rhubarb could inhibit the activation of the NF-*κ*B pathway induced by LPS. It has been reported that NF-*κ*B could be regulated by the PI3K/AKT pathway [23]. These results proved that three species of rhubarb might inhibit the PI3K/AKT/NF-*κ*B signaling pathway, thereby reducing the inflammatory response induced by LPS.

#### 3.8.3. Inhibition of Inflammation-Related Proteins by Three Species of Rhubarb

**Figure 11:**
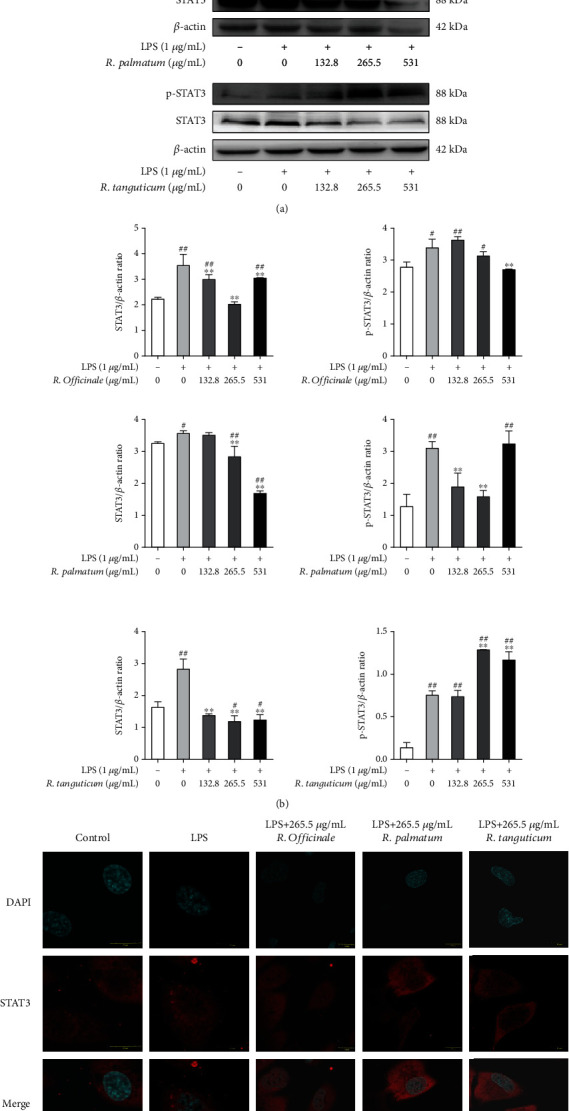
The effect of STAT3 in LPS-induced EA.hy926 cells treated with three species of rhubarb: western blot (a and b) and immunofluorescence (c).

STAT3 and NF-*κ*B are two transcription factors closely linked in the process of inflammation. They share a common target gene and can activate transcription cooperatively [24]. Cytokines encoded by NF-*κ*B, such as IL-6, are both the inflammatory factor encoded by the target gene of NF-*κ*B and the activator of STAT3 [25]. Compared with the LPS model group, western Blot results showed that the expression of STAT3 proteins in EA.hy926 cells was significantly reduced after treatment with three species of rhubarb (*P* < 0.05) (Figure 11). Besides, the expression of p-STAT3 proteins in LPS-induced EA.hy926 cells was significantly reduced after treatment with *R. officinale* high dose and *R. palmatum* low and medium dose (*P* < 0.05) (Figure 11). Immunofluorescence assay results showed that medium doses of rhubarb could slightly inhibit LPS-induced STAT3 phosphorylation nuclear translocation (Figure 11), while the inhibition of p-STAT3 protein expression and nuclear translocation by *R. tanguticum* and *R. officinale* were not obvious and STAT3 might not be the direct target of these two species of rhubarb. The results demonstrated that three species of rhubarb, in especial *R. tanguticum*, could reduce the inflammatory response in LPS-induced EA.hy926 cells by regulating NF-*κ*B nuclear translocation, rather than STAT3, which was consistent with the secretion of inflammatory factors in ELISA experiment.

**Figure 12:**
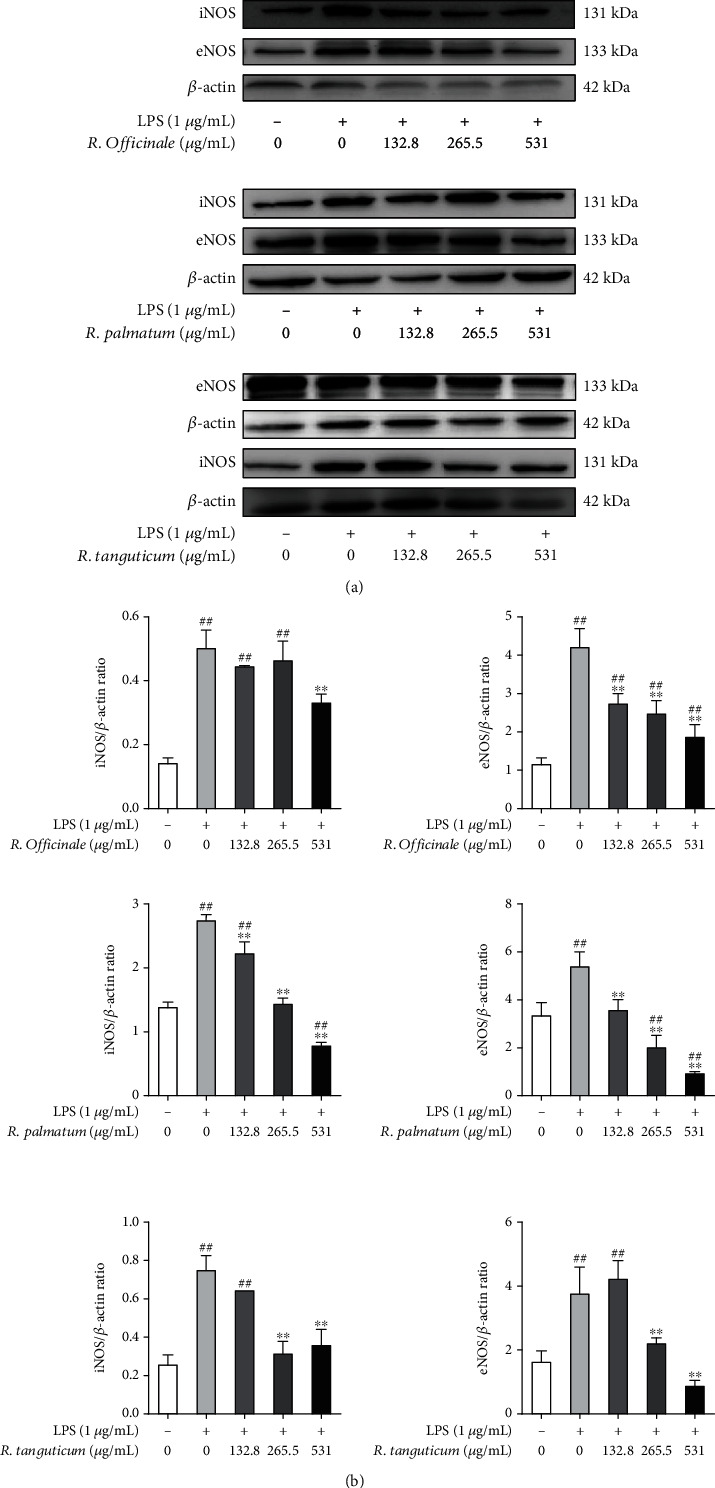
The effect of three species of rhubarb with different concentrations on the protein expressions of NOS in LPS-induced EA.hy926 cells. eNOS (a) and iNOS (b).

NO plays an important role in inflammation and immune response, and excessive production of NO may lead to endothelial injury [26]. eNOS and iNOS are the sources of excess NO in cells [27]. Western blot results showed that the protein expression levels of eNOS and iNOS in LPS model group were significantly increased (*P* < 0.01) and the protein expression levels of eNOS and iNOS were significantly decreased after treatment with three species of rhubarb (*P* < 0.01) (Figure 12). It indicated that three species of rhubarb could inhibit the protein expression of eNOS and iNOS, which was consistent with the experimental results of NO determination.

PI3K/AKT/NF-*κ*B is a classic inflammatory injury pathway of endothelial cells. When activated, the PI3K/AKT/NF-*κ*B pathway can promote the secretion of IL-6, IL-1*β*, TNF-*α*, and other inflammatory factors by downstream related proteins. The continuous accumulation of IL-6 and other inflammatory factors will stimulate STAT3 protein expression and its phosphorylation into the nucleus through pathways such as bypass secretion, promote the expression of inflammation-related genes, and further cause the inflammatory response in the body. At the same time, a large number of inflammatory factors will promote the expression of NOS in cells, resulting in the production of NO in large quantities, which reacts with reactive oxygen species in cells to generate peroxides, directly damaging endothelial cells.

**Figure 13:**
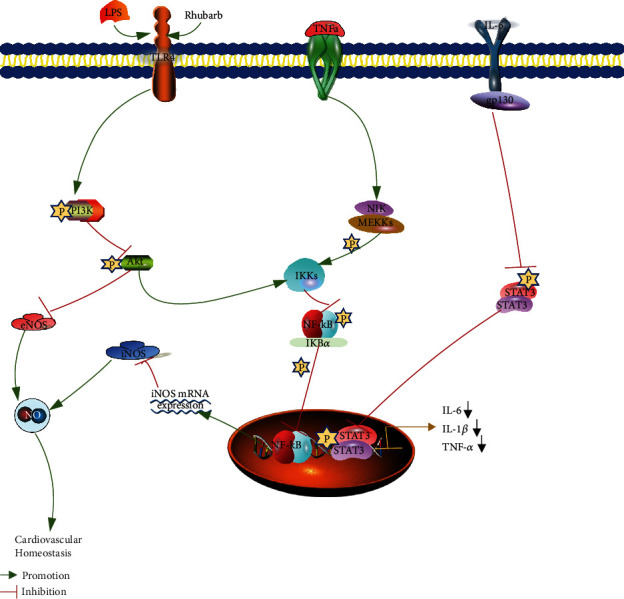
Mechanism of rhubarb inhibiting endothelial injury in EA. hy926 cells induced by LPS.

These results suggested that the three species of rhubarb can reduce the expression levels of NF-*κ*B p65, PI3K, and AKT in LPS-induced EA.hy926 cells; inhibit their phosphorylation; and prevent the activation of PI3K/AKT/NF-*κ*B pathway. The protein expressions of STAT3, eNOS, and iNOS were decreased, and the contents of TNF-*α*, IL-6, IL-1*β*, NO, and ROS in the cells were decreased, which played a role in the inhibition of VEI (Figure 13).

## 4. Discussion

The application of different species as the same TCM was one of the factors that caused the unstable curative effect of TCM. This was likely to be because of genetic differences in these species and the differences in the types, contents, and proportions of their medicinal ingredients, which in turn lead to differences in efficacy. There was still a lack of scientific explanation on whether the use of multiple species as a TCM was reasonable. Therefore, the comparison of different species of TCM had attracted attention in recent years.

The application of plant metabolomics technology to the determination of plant species has been reported in licorice [28], *Bupleurum* [29], *Angelica* [30], and so on. In this paper, the chemical components of three species of rhubarb were analyzed by UPLC-Q-Exactive Orbitrap-MS, and the inhibitory effects of three species of rhubarb on VEI were compared by cell experiments *in vitro*, and 46 effective components of rhubarb were screened out for their inhibitory effects on VEI, including gallic acids and anthocyanins. Gallic acids [4] and anthocyanins [31] were also the material basis of rhubarb on removing blood stasis and dredging meridians.

Through network pharmacology and molecular docking, the potential targets of the effective components of rhubarb in inhibiting VEI were analyzed. The component-target-pathway network diagram was constructed by the existing database, and relatively important targets had a good matching degree with the docking structure of component molecules. AKT1, IL6, STAT3, etc. played an important role in the efficacy of rhubarb, which further illustrated that rhubarb inhibited VEI through “multicomponent, multitarget, and multipathway” [32].

Following the enrichment analysis of GO and KEGG, PI3K/AKT and NF-*κ*B signaling pathways were finally screened out. PI3K/AKT signaling pathway, which played an important role in cell growth cycle, cell migration, cell autophagy, and other life processes [33], had also been involved in the regulation of inflammatory response in recent years [34–36]. It had been reported that phosphorylated PI3K subunits could phosphorylate AKT subunits Thr308 and Ser473 after LPS-induced endothelial cells, which lead to AKT activation [36]. Activated AKT could promote I*κ*B*α* phosphorylation and degradation and activate NF-*κ*B signaling pathways. NF-*κ*B was a classic pathway of inflammation. It had been reported that in inflammatory response, TNF-*α*, IL-6, etc. could activate IKK, which induced the phosphorylation and ubiquitination degradation of the inhibitory protein I*κ*B of NF-*κ*B, transferring NF-*κ*B from the resting state to the nucleus and activating gene transcription [37]. All these indicated that PI3K/AKT could regulate the NF-*κ*B signaling pathway in oxidative responses of endotoxemia [23]. In LPS-induced EA.hy926 cells, the three species of rhubarb showed a good ability to inhibit PI3K, AKT, NF-*κ*B, STAT3, eNOS, iNOS protein expression, and partial protein phosphorylation, exerting anti-inflammatory activity, which was again demonstrated by experimental method that the inflammatory response could be reduced through PI3K/AKT/NF-*κ*B pathway, thus inhibiting VEI. It also illustrated the role of network pharmacology combined with molecular docking in discovering the efficacy of TCM and explaining the mechanism of action.

Generally speaking, the effect of TCM is closely related to the contents of medicinal substances. Even if a medicinal component has a high activity, but with a small content, its pharmacological effect is still weak, and its practical value is low. Therefore, the material basis of this study was found through the correlation between drug efficacy and component contents. However, in this study, only part of index components were screened for inhibiting VEI, and not all. Still some components with high contents and strong activity were ignored. More accurate and comprehensive material basis of rhubarb inhibiting VEI should be further studied. It might be possible to expand the quantitative range of chemical components of rhubarb and associating with pharmacodynamic indicators. Additionally, this study scientifically evaluated the differences in efficacy of the same TCM with different species, which was an important basic research work for the realization of the production of “precision medicinal materials,” and provided theoretical basis for ensuring accurate symptomatic use of TCM.

## 5. Conclusion

In this study, the methods of plant metabolomics, network pharmacology, molecular docking, and cell experiments *in vitro* were combined for the first time to explore the differences of chemical components and inhibition of VEI in rhubarb with three species and clarify the mechanism of rhubarb inhibiting VEI. 36 chemical components were screened in the positive ion mode, and 38 chemical components were screened in the negative ion mode. After the correlation between chemical components and efficacy, 46 effective components were screened to inhibit VEI. *R. tanguticum* had a better inhibitory effect on VEI. Rhubarb inhibited VEI mainly by acting on PI3K, AKT, NF-*κ*B p65, STAT3, eNOS, and iNOS proteins in PI3K/AKT/NF-*κ*B signaling pathway, inhibiting protein expression and phosphorylation, and reducing the contents of TNF-*α*, IL-6, IL-1*β*, NO, and ROS in cells. This study provided an effective way for the determination of precision TCM.

## Figures and Tables

**Table 1:** Different chemical components of three species of rhubarb identified in positive ion mode.

Nol.	R.T. (min)	Mass (*m/z*)	Name	Formula	MS/MS	VIP	*P*
1	21.84	209.0621	3-Hydroxy-3′,4′-Dimethoxyflavone	C_17_H_14_O_5_	[M + H]^+^	1.21	≤0.001
2	26.92	161.0622	4-Methylcoumarin	C_10_H_8_O_2_	[M + H]^+^	1.49	≤0.001
3	10.08	311.0567	5,3′,4′,5′-Tetrahydroxy-6,7-dimethoxyflavone	C_17_H_14_O_8_	M + H-2H_2_O	1.31	≤0.001
4	2.15	158.0820	5,6-Dimethoxynaphtol[2,3-b]furan-4,9-dione	C_14_H_10_O_5_	[M + H]^+^	1.04	≤0.001
5	6.81	233.0453	6-(Carboxymethyl)-7-hydroxy-8-methoxy coumarin	C_12_H_10_O_6_	M + H-H_2_O	1.20	0.01
6	20.06	653.1018	6-Cinnamoyl-1,2-digalloylglucose	C_29_H_26_O_15_	[M + H]^+^	1.37	≤0.001
7	27.02	443.0986	6-Methoxyluteolin-7-glucoside	C_22_H_22_O_12_	M + H-2H_2_O	1.27	≤0.001
8	21.2	255.0691	Chrysophanol	C_15_H_10_O_4_	[M + H]^+^	1.22	≤0.001
9	2.58	1155.2755	Cinnamtannin A_2_	C_60_H_50_O_24_	[M + H]^+^	1.15	≤0.001
10	3.61	189.0556	cis-Sinapic acid	C_11_H_12_O_5_	M + H-2H_2_O	1.26	≤0.001
11	14.24	727.1226	Cy 3-coumSamb-5-Glc	[C_41_H_45_O_22_]^+^	[M + H]^+^	1.43	≤0.001
12	7.76	882.1615	Emodin	C_15_H_10_O_5_	[M + H]^+^	1.18	≤0.001
13	4.65	731.1593	ent-Epicatechin-(4alpha->8)-ent-epicatechin 3-gallate	C_59_H_46_O_26_	[M + H]^+^	1.24	0.02
14	27.79	239.0714	(-)-Epiafzelechin	C_15_H_14_O_5_	M + H-2H_2_O	1.07	≤0.001
15	6.55	151.0391	(-)-Epicatechin	C_15_H_14_O_6_	[M + H]^+^	1.26	≤0.001
16	3.61	289.0716	Epigallocatechin	C_15_H_14_O_7_	M + H-H_2_O	1.20	0.03
17	6.53	441.0827	(-)-Epigallocatechin gallate	C_22_H_18_O_11_	M + H-H_2_O	1.16	≤0.001
18	4.63	577.1357	Epigallocatechin-(4beta- >8)-catechin	C_30_H_26_O_13_	M + H-H_2_O	1.01	0.02
19	27.02	125.0254	Ethyl vanillate	C_10_H_12_O_4_	[M + H]^+^	1.25	≤0.001
20	19.13	191.0359	Glycyrrhetinic acid	C_30_H_46_O_4_	[M + H]^+^	1.22	≤0.001
21	21.48	547.1599	Guibourtinidol-(4alpha->6)-catechin	C_30_H_26_O_10_	[M + H]^+^	1.12	≤0.001
22	2.78	517.1560	Luteone 4′,7-*O*-diglucoside	C_27_H_30_O_16_	[M + H]^+^	1.09	0.01
23	3.53	369.0294	Mangiferin	C_19_H_18_O_11_	[M + H]^+^	1.29	≤0.001
24	0.67	113.0241	Mesaconic acid	C_5_H_6_O_4_	[M + H]^+^	1.14	≤0.001
25	5.58	189.0562	Myricetin	C_15_H_10_O_8_	[M + H]^+^	1.12	≤0.001
26	5.07	439.1606	Neomangiferin	C_25_H_28_O_16_	[M + H]^+^	1.69	≤0.001
27	7.77	893.1569	Oolonghomobisflavan A	C_45_H_36_O_22_	M + H-2H_2_O	1.12	≤0.001
28	18.30	464.1272	Peonidin-3-*O*-beta-*D*-glucoside	[C_22_H_23_O_11_]^+^	[M + H]^+^	1.22	≤0.001
29	4.65	189.0574	Plumbagin	C_11_H_8_O_3_	[M + H]^+^	1.06	0.01
30	3.84	1462.3159	Procyanidin B-2,3,3′-di-*O*-gallate	C_44_H_34_O_20_	[M + H]^+^	1.24	≤0.001
31	4.38	183.0821	Resveratrol	C_14_H_12_O_3_	[M + H]^+^	1.00	0.001
32	21.84	255.0692	Rhein	C_15_H_8_O_6_	[M + H]^+^	1.20	≤0.001
33	4.89	867.2121	Robinetinidol-(4alpha->8)-catechin-(6->4alpha)-robinetinidol	C_45_H_38_O_18_	[M + H]^+^	1.17	≤0.001
34	22.17	549.1775	Sachaliside 2	C_30_H_32_O_12_	M + H-2H_2_O	1.15	≤0.001
35	18.64	880.1926	Sennoside A	C_42_H_38_O_20_	[M + H]^+^	1.07	≤0.001
36	4.65	679.0703	Torachrysone 8-glucoside	C_20_H_24_O_9_	[*M* + *H*]^+^	1.05	≤0.001

**Table 2:** Different chemical components of three species of rhubarb identified in negative ion mode.

Nol.	R.T. (min)	Mass (*m/z*)	Name	Formula	MS/MS	VIP	*P*
1	7.56	566.1958	4-(4-Hydroxyphenyl)-2-butanone *O*-[2,6-digalloylglucoside]	C_30_H_30_O_15_	[M-H]^−^	1.15	≤0.001
2	28.32	205.1603	(-)-Epicatechin	C_15_H_14_O_6_	[M-H]^−^	1.20	≤0.001
3	6.53	886.1869	(-)-Epicatechin gallate	C_22_H_18_O_10_	[M-H]^−^	1.24	≤0.001
4	15.05	939.1112	Pentagalloylglucose	C_41_H_32_O_26_	[M-H]^−^	1.21	≤0.001
5	19.17	255.0692	5,8-Dihydroxyflavanone	C_15_H_12_O_4_	[M-H]^−^	1.12	≤0.001
6	4.73	390.1415	7,12-Dioxo-5a-cholan-24-oic acid	C_24_H_36_O_4_	[M-H]^−^	1.11	≤0.001
7	2.32	189.0554	7-Ethoxycoumarin	C_11_H_10_O_3_	[M-H]^−^	1.05	0.02
8	10.08	311.0567	Aloe emodin	C_17_H_12_O_6_	[M-H]^−^	1.46	0.01
9	17.36	609.1686	Apiin	C_26_H_28_O_14_	[M-H]^−^	1.14	≤0.001
10	6.94	951.1323	Cassiaside	C_20_H_20_O_10_	[M-H]^−^	1.23	≤0.001
11	6.11	883.1701	Catechin gallate	C_22_H_18_O_10_	[M-H]^−^	1.24	≤0.001
12	27.79	239.0714	Chrysarobin	C_15_H_12_O_3_	[M-H]^−^	1.07	≤0.001
13	22.68	239.0713	Chrysophanic acid 9-anthrone	C_15_H_12_O_3_	[M-H]^−^	1.35	0.02
14	11.07	729.1464	Ent-Epicatechin-(4alpha->8)-ent-epicatechin 3-gallate	C_37_H_30_O_17_	[M-H]^−^	1.25	≤0.001
15	3.64	273.0768	(-)- (*2R*,*3R*) Epiafzelechin	C_15_H_14_O_5_	[M-H]^−^	1.03	0.02
16	19.63	191.0715	Ethyl-p-coumarate	C_11_H_12_O_3_	[M-H]^−^	1.11	0.04
17	20.04	443.0992	Formononetin 7-*O*-glucuronide	C_21_H_18_O_10_	[M-H]^−^	1.11	0.02
18	7.52	125.0251	Gallic acid	C_7_H_6_O_5_	[M-H]^−^	1.16	≤0.001
19	1.32	602.0134	Gallic-acid-*O*-galloyl-glucoside	C_20_H_20_O_14_	[M-H]^−^	1.13	≤0.001
20	26.92	161.0622	Glabranine	C_20_H_20_O_4_	[M-H]^−^	1.34	≤0.001
21	28.20	255.0662	Isoliquiritigenin	C_15_H_12_O_4_	[M-H]^−^	1.29	0.02
22	5.58	189.0562	Kaempferol	C_15_H_10_O_6_	[M-H]^−^	1.02	≤0.001
23	8.04	835.2391	Liquiritin	C_21_H_22_O_9_	[M-H]^−^	1.11	≤0.001
24	6.78	575.1085	Procyanidin B_1_	C_30_H_26_O_12_	[M-H]^−^	1.06	≤0.001
25	2.58	1155.2755	Procyanidin B_2_	C_30_H_26_O_12_	[M-H]^−^	1.12	0.03
26	15.03	945.1180	Procyanidin B-2,3,3′-di-*O*-gallate	C_44_H_34_O_20_	[M-H]^−^	1.23	≤0.001
27	4.67	730.1512	Procyanidin B_4_	C_30_H_26_O_12_	[M-H]^−^	1.29	≤0.001
28	4.67	729.1479	Procyanidin B_5_	C_30_H_26_O_12_	[M-H]^−^	1.30	≤0.001
29	8.82	727.0850	Procyanidin B-5,3,3′-di-*O*-gallate	C_44_H_34_O_20_	[M-H]^−^	1.03	≤0.001
30	4.89	867.2121	Procyanidin C_1_	C_45_H_38_O_18_	[M-H]^−^	1.13	0.01
31	5.05	440.1660	Prunin	C_21_H_20_O_11_	[M-H]^−^	1.39	≤0.001
32	22.17	549.1775	Quercetin-3-(6^″^-malonyl)-glucoside	C_24_H_22_O_15_	[M-H]^−^	1.18	≤0.001
33	11.23	462.1145	Quercetin-3,4′-*O*-di-beta-glucopyranoside	C_27_H_30_O_17_	[M-H]^−^	1.10	0.01
34	27.06	610.2032	Vincetoxicoside A	C_27_H_30_O_16_	[M-H]^−^	1.24	≤0.001
35	6.09	980.1351	Resveratrol-4′-*O*-(6^″^-galloyl)-b-d-glucopyranoside	C_27_H_26_O_12_	[M-H]^−^	1.03	≤0.001
36	19.37	696.1174	Sennoside A	C_42_H_38_O_20_	[M-H]^−^	1.06	≤0.001
37	28.22	247.1000	Torachrysone 8-glucoside	C_20_H_24_O_9_	[M-H]^−^	1.07	≤0.001
38	3.13	179.0350	Trans-2,3-Dihydroxycinnamate	[C_9_H_7_O_4_]^−^	[M-H]^−^	1.08	0.01

**Table 3:** Comprehensive efficacy of three species of rhubarb in inhibiting VEI.

Group	Relative content of NO	Relative content of ROS	Relative content of TNF-*α*	Relative content of IL-6	Relative content of IL-1*β*	Comprehensive efficacy (I)
Low-dose group of *R. officinale*	1.2917	0.7501	0.3423	0.6198	0.7208	0.7853
Medium-dose group of *R. officinale*	0.6183	0.1812	0.3681	0.5293	0.5082	0.4862
High-dose group of *R. officinale*	0.2917	0.0493	0.9600	0.4582	0.4520	0.4636
Low-dose group of *R. palmatum*	0.0477	0.0483	0.3600	0.9026	0.6822	0.4536
Medium-dose group of *R. palmatum*	0.2954	0.0007	0.4401	0.6986	0.4968	0.4371
High-dose group of *R. palmatum*	0.9835	0.3938	0.4205	0.4996	0.2912	0.5628
Low-dose group of *R. tanguticum*	0.4220	0.0095	0.3904	1.0555	0.3932	0.5341
Medium-dose group of *R. tanguticum*	0.3761	0.2556	0.3392	0.7934	0.1826	0.4298
High-dose group of *R. tanguticum*	1.3927	0.6567	0.3229	0.3280	0.7099	0.7197

**Table 4:** Relative correlation between the potential effective components of three species of rhubarb and comprehensive efficacy of inhibiting VEI.

Nol.	Name	Relative correlation	Nol.	Name	Relative correlation
1	Gallic acid	0.8153	24	Mesaconic acid	0.7291
2	Gallic-acid-*O*-galloyl-glucoside	0.7916	25	Pentagalloylglucose	0.7282
3	Procyanidin B-2,3,3′-di-*O*-gallate	0.7750	26	Formononetin 7-*O*-glucuronide	0.7279
4	Peonidin-3-*O*-beta-*D*-glucoside	0.7728	27	Plumbagin	0.7270
5	Procyanidin B-5,3,3′-di-*O*-gallate	0.7718	28	Ethyl vanillate	0.7258
6	Cassiaside	0.764	29	(-)-Epicatechin gallate	0.7246
7	Glabranine	0.7635	30	4-Methylcoumarin	0.7237
8	Mangiferin	0.7632	31	Torachrysone 8-glucoside	0.7236
9	5,6-Dimethoxynaphtol[2,3-b]furan-4,9-dione	0.7628	32	(-)-Epicatechin	0.7231
10	Guibourtinidol-(4alpha->6)-catechin	0.7627	33	Apiin	0.7212
11	Resveratrol-4′-*O*-(6^″^-galloyl)-b-d-glucopyranoside	0.7555	34	Procyanidin B_2_	0.7199
12	Resveratrol	0.7553	35	5,8-Dihydroxyflavanone	0.7174
13	Ent-Epicatechin-(4alpha->8)-ent-epicatechin 3-gallate	0.7529	36	Chrysophanol	0.7170
14	7,12-Dioxo-5a-cholan-24-oic acid	0.7518	37	Procyanidin B_1_	0.7115
15	Cinnamtannin A_2_	0.7511	38	Catechin gallate	0.7113
16	Cy 3-coumSamb-5-Glc	0.7499	39	3-Hydroxy-3′,4′-Dimethoxyflavone	0.7100
17	Oolonghomobisflavan A	0.748	40	Glycyrrhetinic acid	0.7092
18	6-(Carboxymethyl)-7-hydroxy-8-methoxy Coumarin	0.7474	41	Aloe emodin	0.7086
19	Sennoside A	0.7443	42	Vincetoxicoside A	0.7071
20	Epigallocatechin	0.7409	43	Cis-Sinapic acid	0.7065
21	Procyanidin C_1_	0.7401	44	Quercetin-3,4′-*O*-di-beta-glucopyranoside	0.7054
22	Emodin	0.7354	45	5,3′,4′,5′-Tetrahydroxy-6,7-dimethoxyflavone	0.7047
23	6-Cinnamoyl-1,2-digalloylglucose	0.7325	46	Neomangiferin	0.7004

## Data Availability

The data used to support the findings of this study are available from the corresponding author upon request.
